# Improving *Tenebrio molitor* Growth and Nutritional Value through Vegetable Waste Supplementation

**DOI:** 10.3390/foods13040594

**Published:** 2024-02-16

**Authors:** Gloria López-Gámez, Raquel del Pino-García, María Asunción López-Bascón, Vito Verardo

**Affiliations:** 1 Research and Development of Functional Food Center (CIDAF), Avda. del Conocimiento, 37, 18016 Granada, Spain; rdpinogarcia@cidaf.es (R.d.P.-G.); alopez@cidaf.es (M.A.L.-B.); vitoverardo@ugr.es (V.V.); 2 Department of Nutrition and Food Science, Campus of Cartuja, University of Granada, 18071 Granada, Spain; 3 Institute of Nutrition and Food Technology Jose Mataix, Biomedical Research Center, University of Granada, Avda. Conocimiento s/n, 18100 Granada, Spain

**Keywords:** protein alternative, circular economy, fatty acids, insect growth, vegetable waste, diet supplementation

## Abstract

Huge amounts of vegetable wastes are generated by the food industry. Their bioconversion into valuable products (e.g., insect flours or biofertilizer) through insect farming is a promising solution to reduce their negative environmental and economic impacts. This study evaluates the growth of *Tenebrio molitor* larvae and their nutritional profile after supplementing their diets with vegetable wastes. Over a 6-week period, 45-day larvae were fed a diet comprising wheat bran supplemented (1:1) with cucumber or tomato wastes from both conventional and ecological crops. The control diet consisted of wheat bran and an equivalent amount of water to compensate for the waste moisture. Larval weight was measured weekly, and length measures were taken fortnightly. Nutritional composition and fatty acid profile were analyzed at the end of the study in 90-day larvae. Regardless of using vegetable waste from conventional or ecological harvesting, the weight of 6-week supplemented larvae almost doubled that of larvae fed with just wheat bran, and their length was 15% higher. Supplementation also increased larval polyunsaturated fatty acid percentage by 22–37%, with linoleic acid being the most abundant. Likewise, larval protein content reached 50% after supplementation. This study demonstrates that both cucumber and tomato wastes from conventional or ecological crops are excellent supplements for *T. molitor’s* diet, improving their nutritional value and reducing the time necessary for larvae growth.

## 1. Introduction

According to the Food and Agriculture Organization of the United Nations [1] and the United Nations Department of Economic and Social Affairs [2], the demand for animal protein is projected to double by 2050 due to the growing global population. It is important to note that the meat production industry is widely recognized for its significant environmental impact [3]. In light of this challenge, food scientists and the industry are exploring innovative approaches. One proposed solution involves reducing or replacing meat consumption with alternative protein sources such as insects. The use of insects as an alternative food holds great promise on a global scale, not only because of their nutritional value (high-quality protein, polyunsaturated fats, vitamins, and minerals) but also due to their cost-effectiveness and environmentally friendly characteristics [4,5].

The concept of the circular economy has gained global prominence, aiming to establish a sustainable food system that prioritizes food quality, safety, and environmental conservation [6]. In recent times, insect-based bioconversion has emerged as a promising strategy for revaluing agri-food waste and contributing to the development of a circular economy [7]. This innovative approach capitalizes on the capacity of insects to convert large quantities of food waste into valuable products for both animal and human nutrition, as well as generating frass suitable for use as organic biofertilizer in ecological crops [8]. Additionally, insect farming has environmental benefits, including minimal ammonia and greenhouse gas emissions [5], low water footprint [9], and reduced land usage [10]. Among the most used insects for bioconversion, *Tenebrio molitor* stands out, given its ability to adapt its larval development period, weight, and nutritional composition depending on its diet. While mealworms can primarily subsist on a diet based on wheat bran, they are commonly nourished with additional vegetable materials to enhance their nutrient intake [10]. Previous research has examined the effects of dietary supplementation on mealworms, including aspects such as their survival, growth, feed conversion, and nutrient composition [11,12,13,14]. Nevertheless, a limited number of studies have explored supplements rich in antioxidants, vitamins, and minerals. Insects are susceptible to oxidative stress, which can detrimentally affect their growth and development as well as their nutritional composition [15]. Therefore, while typically *T. molitor* is fed with grains, supplementing or partially substituting their diet with vegetable wastes presents a cost-saving alternative that reduces the use of resources intended for human consumption and minimizes environmental impact. Furthermore, vegetable waste, being rich in water content and bioactive compounds such as phenolic compounds, terpenoids, vitamins, and sulfur compounds, not only may promote insect growth but may also contribute health-enhancing properties to insect-based products [16].

Mealworms are also nutritionally interesting for their fatty acid composition, which can be influenced by the feed they consume, ultimately contributing to a well-balanced human diet. Fatty acids contribute to the production of hormones and antibodies, provide energy, and participate in a variety of cellular processes [17]. The human body synthesizes most of these fatty acids, such as saturated fatty acids (SFAs), whereas essential polyunsaturated acids (PUFAs) like linoleic and α-linolenic acids must be obtained through dietary intake. Such PUFAs are precursors for omega-6 and omega-3 fatty acids and can be found in *T. molitor* [18].

In the southern regions of Spain, significant amounts of cucumbers and tomatoes are being discarded in greenhouses and sorting facilities by marketers due to non-compliance with quality standards. While most of these vegetable wastes come from conventional cultivation, there is a rising proportion contributed by ecological crops. These ecological crops may contain elevated levels of phytochemicals, acting as a natural defense mechanism against environmental stressors.

Therefore, the main objective of this study was to assess the impact of supplementing the diet of *T. molitor* larvae with vegetable waste, specifically cucumber and tomato waste from both conventional and ecological crops, on their growth performance, feed conversion efficiency, and fatty acid composition from a sustainable approach.

## 2. Materials and Methods

### 2.1. Insect Diet

Insect farming was conducted at Insectalia S.L. (Madrid, Spain). *T. molitor* larvae were provided with 5 different diets, including a control diet of wheat bran (referred to as “W”), or diets supplemented in a 1:1 ratio with vegetable waste: cucumber (referred to as “C + W”) or tomato (referred to as “T + W”). These vegetable wastes were sourced from both conventional (C) and ecological (E) harvesting and were kindly provided by Grupo La Caña company (Puntalón, Granada, Spain). Nutrient composition, vitamins, and bioactive compounds of each commodity can be reviewed in Table 1 and Table 2, respectively. To maintain similar moisture levels among diets, water was added by spraying the control diet with the same quantity of water as contained the vegetable wastes.

### 2.2. Insect Farming

*T. molitor* larvae were grown in trays placed in a controlled chamber room with 12 h light–dark cycles, and maintained at 27 °C and 50% humidity. During an initial period of around 6 weeks (45 days), all larvae were fed with the control diet (W). Then, larvae were sieved to separate frass and divided into 5 groups ((C + W) C; (C + W) E; (T + W) C; (T + W) E; or W). Each diet condition was studied using 5 trays, each initially containing 100 g of the 45-day larvae. Then, the larvae were fed with the corresponding diet once a week for a total of 6 weeks, until 90-day larvae were collected at the end of the study (before supplemented larvae began to pupate).

During the first three weeks of supplementation, 150 g of wheat bran and 150 g of the corresponding vegetable waste were added to each tray. In the fourth week, this quantity was increased to 200 g per tray, and in the fifth and sixth weeks, 400 g per tray was added. Therefore, at the end of the study, larvae were provided 1.4 kg of the corresponding vegetable waste and 1.4 kg of wheat bran, whereas the control larvae received 1.4 kg of wheat bran and 1.4 kg of added water (Table 3). Vegetable wastes were sliced before being supplemented to ensure larvae could eat them better. After the 6-week period, the larvae were subjected to a 48 h starvation period, collected, and then frozen at −20 °C.

### 2.3. Growth Performance Measurement

To assess the growth performance of the larvae, each tray was sieved to separate the frass from the larvae. The total weight of the larvae was measured weekly using a precision balance for each tray, whereas the unitary weight was measured as the average of 5 larvae per tray (25 larvae/group). The length of the larvae was measured every two weeks using a vernier caliper, with the measurement taken as the average of 5 larvae per tray. Subsequently, the quantities of wheat bran plus water or vegetable waste required for each week were added as stated in Section 2.2.

The survival rate, feed conversion ratio (FCR), the efficiency of conversion of ingested feed (ECI), and nitrogen conversion efficiency (N-ECI) were calculated according to the following equations [11,18]:

(1) $$FCR=\frac{weight\;of\;ingested\;food}{weight\;gained}$$

(2) $$ECI=\frac{final\;weight}{weight\;of\;ingested\;feed}\times 100$$

(3) $$N-ECI=\frac{insect\;N\;content\times insect\;weight\;at\;harvest}{dietary\;N\;content\times feed\;provided}$$

### 2.4. Macronutrient Composition Analysis

Macronutrient composition analyses were carried out in commodities used for formulating the diets and in the resulting adult larvae after finishing the rearing. Vegetable wastes were sliced and frozen at −20 °C for 1 h. Thereafter, they were freeze-dried at 21.2 °C and 0.02 mbar for 7 days. Dried wastes were vacuum-sealed and stored at room temperature until their analysis. On the other hand, frozen larvae were used to measure moisture and crude fat, whereas crude protein and ash were determined in larvae flour, obtained after infrared oven drying at 68 °C for 4.5 h. The analysis of moisture, crude protein content, crude fat content, ash content, and carbohydrate content was outsourced to a certified nutritional analysis laboratory.

### 2.5. Fatty Acid Profile

For the determination of fatty acids, an extraction and derivatization process was carried out. The fatty acid methyl esters (FAMEs) were obtained following the AOAC official method 996.06 [19]. Extraction and derivatization were carried out in triplicate.

The samples were analyzed by an Agilent 7890B gas chromatograph coupled to a 7200 quadrupole time-of-flight mass spectrometer with electron impact ionization (Agilent Technologies, Santa Clara, CA, USA). The FAMEs were separated using an HP-88 capillary column (30 m, ø 0.25 mm, and a film thickness of 0.2 μm), with ultrapure grade helium used as the carrier gas at a flow rate of 1 mL/min. A 1 µL sample was injected with a split ratio of 1:20 (*v*/*v*). The oven temperature was programmed to increase from 80 °C to 145 °C at a rate of 8 °C/min, maintaining this temperature for 26 min, then increasing to 200 °C at a rate of 2 °C/min and holding it for an additional minute, and finally ramping up to 220 °C at a rate of 8 °C/min. A solvent delay of 2 min was used to prevent damage in the ion source filament. The injector and transfer line temperatures were kept at 250 and 240 °C, respectively. The TOF detector’s conditions were as follows: ionization source temperature of 230 °C, ionization energy of 70 eV, mass range of 50–500 *m*/*z.* daily mass calibration was performed with PFTBA. MassHunter GC QTOF Acquisition software (version B.06, Agilent Technologies) was used to control data acquisition and set the parameters for optimum operation. Tentatively identified FAMEs were confirmed by comparing them with a mixture of 37 FAMEs from Supelco (Merck, Madrid, Spain). Qualitative MassHunter software (version B.06, Agilent Technologies, Santa Clara, CA, USA) was used for data processing. The concentration of fatty acids was expressed as percentage (%).

In addition, the main nutritional indices related to fatty acid and oxidizability (COX) values were calculated by using the fatty acid profile of *T. molitor* larvae [20]. The nutritional indices used were PUFA/SFA ratio, index of atherogenicity (IA), index of thrombogenicity (IT), hypocholesterolemic/hypercholesterolemic (HH) ratio, and health-promoting index (HPI). The indices were calculated according to Kotsou et al. [16].

### 2.6. Statistical Analysis

Statistical analyses were conducted using the Statgraphics Centurion XVI.I software version 16.1.18 from Statgraphics Technologies Inc. (The Plains, Virginia, USA). The results are presented as the mean ± standard deviation. An analysis of variance (ANOVA) was performed, followed by a Tukey post hoc test to determine statistical differences among the mean values. The significance level for statistical tests was set at *p* < 0.05.

## 3. Results and Discussion

### 3.1. Diet Influence on Growing Parameters of T. molitor Larvae

**Figure 1:**
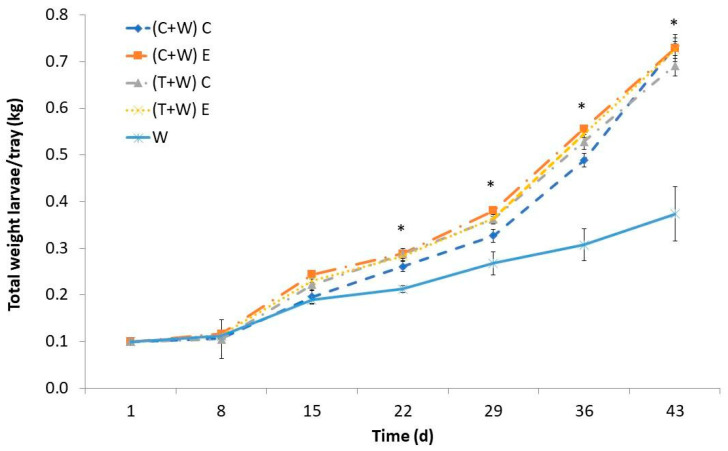
Larval weight of *T. molitor* determined weekly while feeding for 43 days with different diets [(C + W) C: cucumber waste + wheat bran, conventional crop; (C + W) E: cucumber waste + wheat bran, ecological crop; (T + W) C: tomato waste + wheat bran, conventional crop; (T + W) E: tomato waste + wheat bran, ecological crop; W: wheat bran]. Asterisks (*) indicate statistical difference (*p* < 0.05) between the larval weight of those fed with W (control diet) and that of those supplemented with vegetable wastes at the same rearing time. Values are expressed as mean ± standard deviation (*n* = 5).

Over time, there was a noticeable increase in the weight of *T. molitor* larvae, with the most significant growth occurring between the 15th and 43rd days. The larval weight was influenced by the provided diet, and noteworthy differences were significant between the groups that were supplemented and the control group starting from the 22nd day (Figure 1). Interestingly, regardless of the harvesting (conventional or ecological) and the type of vegetable waste (cucumber or tomato), there were no significant differences in the larval weight. Larvae solely fed wheat bran exhibited notably lower weights than their counterparts that received vegetable waste supplements. By the 43rd day, larvae fed with cucumber or tomato waste, regardless of harvesting, reached weights ranging from 0.647 to 0.720 kg per tray, nearly doubled the weight of larvae fed only wheat bran (0.370 kg per tray). Consequently, supplementation significantly promoted larval growth, leading to a sevenfold increase in weight from the study outset. In contrast, the weight of larvae fed exclusively with wheat bran increased by less than fourfold. All in all, this finding means that supplementing with cucumber or tomato wastes, both from conventional and ecological crops, decreases farming time of *T. molitor* larvae by 3–4 weeks.

**Figure 2:**
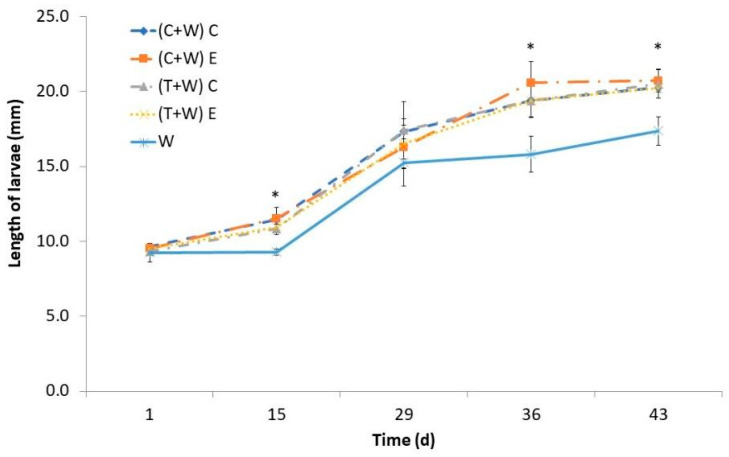
Larval length of *T. molitor* determined every fortnight while feeding for 43 days with different diets [(C + W) C: cucumber waste + wheat bran, conventional crop; (C + W) E: cucumber waste + wheat bran, ecological crop; (T + W) C: tomato waste + wheat bran, conventional crop; (T + W) E: tomato waste + wheat bran, ecological crop; W: wheat bran]. Asterisks (*) indicate statistical difference (*p* < 0.05) between the larval length of those fed with wheat bran (control diet) and that of those supplemented with vegetable wastes at the same rearing time. Values are expressed as mean ± standard deviation (*n* = 5).

The length of the larvae exhibited a gradual increase over time, with a more pronounced growth phase starting from the 15th day. The larval length was also influenced by their diet, and noticeable disparities became apparent between the groups receiving vegetable supplementation and the control diet (just wheat bran) after the 36th day (Figure 2). However, the length of larvae supplemented with agricultural wastes was similar regardless of the kind of waste (cucumber or tomato) and the type of harvesting (conventional or ecological). By day 43, larvae fed with vegetable waste attained a larger size of approximately 20 mm, surpassing those fed the control diet, which measured around 17 mm.

Similar reduction in growing time has been reported when vegetable by-products were provided as a supplement [21,22,23], which can be due to their water content, since the growing performance of *T. molitor* is strongly affected by its availability [24]. For instance, Urs et al. [25] observed higher larval weight and length when water cotton pads on a free-choice basis were available. Some authors attributed the higher larval weight to lipid accumulation, which could be used to obtain energy for their adult life [25]. However, obtained results regarding larval total fat content demonstrated that lipids were not more accumulated in larvae fed with vegetable supplements than in larvae fed just with wheat bran (Table 4). Therefore, the increased weight may be linked to the higher water content in the larvae. Water can be assimilated by mealworm larvae by absorption through cuticles or by ingestion when available [25]. Our results demonstrated that ingestion of water embedded in vegetables instead of absorption could be beneficial to speed larval development. Also, the increased consumption of micronutrients and bioactive compounds (Table 2) of supplemented larvae could influence their metabolism and result in accelerated growing. Similar results of larval weight have been reported when *T. molitor’s* diet was supplemented with *Moringa oleifera* leaves [16] or orange albedo [26], both sources of polyphenols, carotenoids, and vitamins. In addition, it has been shown that the inclusion of carrot pomace, rich in carotenoids, in *T. molitor’s* diet also favored their growing by reducing the oxidative stress in insects [22].

### 3.2. Diet Influence on T. molitor Larvae’s Nutritional Value

Nutritional composition and moisture content of *T. molitor* larvae depended on the provided diet (Table 4). The moisture content of the supplemented larvae was 14.93–18% higher than that contained in larvae fed just wheat bran (Table 4). Although the larvae supplemented with conventional tomato had a significantly higher moisture content than the rest of the supplemented larvae, these differences were not correlated with differences in the contents of nutritional or bioactive compounds of different wastes. The obtained results support the hypothesis formulated about the better assimilation of water contained in the agricultural wastes by larvae than that added to the wheat bran control diet. The accumulation of water in the larval body can contribute to improving the assimilation of nutrients, which ultimately led to an increase in the growth of the supplemented larvae (Figure 1 and Figure 2) [27]. Similar results regarding higher mealworm growth were reported when vegetable wastes (carrot pomace) were provided as a moisture source in the diet [22].

The total protein content was notably increased in the larvae that were supplemented with cucumber and tomato wastes, regardless of conventional or ecological sources, as shown in Table 4. A similar outcome was observed in a study by Ruschioni et al. [21] when they supplemented the larvae diet with olive pomace, which had a protein content similar to that of the control diet (ranging from 11 to 15% on a dry weight basis). There has been controversial debate over the impact of diet on the protein content of larvae. Oonincx et al. [11] have hypothesized that high-protein diets result in *T. molitor* larvae with higher protein content. Some authors have studied the correlation between dietary protein content in substrates and protein level in larvae and concluded that the correlation was significant but not strong enough to elucidate this issue (R^2^ = 0.573 [28], R^2^ = 0.3169 [29], and R^2^ = 0.36 [30]). In contrast, Van Broekhoven et al. [12] demonstrated that larvae maintained similar protein content, even when their diets differed in protein content by two to threefold. Therefore, other factors during rearing and macronutrients present in the diet can also affect protein content in larvae. For instance, Zhang et al. [31] obtained larvae with increased protein and carbohydrate contents when they were fed with fiber-rich diets (highly denatured soybean meal, mushroom spent corn stover, and spirit distillers’ grains) instead of basing their diet on wheat bran. However, Kröncke et al. [28] found a weak correlation (R^2^ = 0.432) between carbohydrate content in the diet and protein levels in larvae. Similarly, Jajić et al. [29] also reported a regression coefficient of R^2^ = 0.5711 between the starchy carbohydrate content in substrates and protein levels in larvae. Therefore, a clear relationship cannot be established between the consumed carbohydrate content and the protein content found in larvae. According to the obtained results and the controversial information found in the literature surrounding this issue, it has been proposed that the excess of dietary protein may be excreted in the form of uric acid through feces [12]. In our study, the primary source of dietary protein was wheat bran, constituting approximately 15% of its fresh weight, which was 21 and 14 times higher than in cucumber and tomato wastes, respectively. To obtain 1 kg of larvae, the total amount of protein provided to the larvae in their diet was approximately 0.87 kg for C + W, 0.92 kg for T + W, and 1.2 kg for W. This suggests that the (C + W) C and (C + W) E diets were the most effective supplements for larvae to convert dietary protein into their own protein. However, further research is needed to unravel the effects of diet composition on larval metabolism, growth, and composition.

Carbohydrate content in larvae was also affected by the provided diet (Table 4); for instance, the total content was significantly reduced by 12.6–21.4% when tomato waste was used as a supplement. As far as we know, there are no available studies in which the carbohydrate content of *T. molitor* larvae after supplementation is evaluated. Some authors have reported that larvae adapt their metabolism to the provided diet, and a high level of starch can reduce their growth [32], as observed in our research, since the growth of larvae fed with just wheat bran was lower than that of supplemented larvae, whose diet had a slightly lower percentage of starch and more fiber. Both the quantity and type of carbohydrate or fiber that larvae consume exert an influence on their growth [28]. The proportion of cellulose, hemicellulose, and lignin as well as the type of carbohydrates that compose wheat bran and vegetable wastes is different. Hence, the higher growing rate could be explained by the higher digestibility of vegetable starch [12], which is used preferentially to obtain energy rather than to be accumulated in tissues for further use. Regarding the larval carbohydrate content, some authors have reported a weak correlation between the nutrients in substrates and those found in larvae [29,30]. Observed differences are probably related to modulations in larval metabolism, which is adapted to the provided nutrients, although further studies are necessary to understand such changes.

The total fat content differed among larvae fed with supplemented diets or just wheat bran (Table 4). Supplementing diets with cucumber wastes ((C + W) C and (C + W) E) reduced the fat content in larvae by 12.41–13.48%, whereas larvae fed with tomato wastes ((T + W) C and (T + W) E) showed similar fat content to the control group. Insects can synthesize lipids from different dietary components, such as carbohydrates [12], which might explain the reduced carbohydrate content and the increased total lipid content in those larvae supplemented with tomato wastes (Table 4). Some authors have studied the influence of diet composition on fat content, although no conclusive results have been reached yet. Some studies have demonstrated that fat content remained constant despite provided substrates having different fat content [33], while other authors have found that the total content and the fatty acid profile was influenced by the fat composition of the feed source [12,33]. Besides, weak correlations have been found between the protein and carbohydrate contents in diets and fat content found in larvae, with R^2^ = 0.253 and R^2^ = 0.332, respectively [28]. Carbohydrates and lipids are mainly used for energy, while protein is most efficiently utilized for tissue growth [28]. This fact may explain the variations in the macronutrient content of the larvae based on the diet provided and growth needs. However, these differences can be better explained from variations in the fatty acid profile.

The primary fatty acids identified in *T. molitor* larvae are presented in Table 5, of which most are in accordance with those reported by other authors [31,34]. Their abundance, from highest to lowest, follows this order: linoleic acid > elaidic acid > myristic acid = palmitic acid > stearic acid. The findings reveal that the fatty acid composition and the ratio of polyunsaturated to saturated fatty acids are influenced by the provided diet, a pattern consistent with previous research [33,34,35]. Likewise, the storage of polyunsaturated fatty acids has been reported in other species such as *Zophobas atratus* [36]. Supplementing the larval diet with cucumber and tomato waste, regardless of the type of harvesting, resulted in an increased linoleic acid content, ranging from 6.42% to 31.9% more than in the larvae fed with the control diet, although contrary results were reported when larvae were supplemented with *M. oleifera* [16]. The highest linoleic acid content reached 11.1 g/100 g larvae, and it was achieved when the diet was supplemented with tomato waste from ecological harvesting (Table 5). Furthermore, larvae fed diets supplemented with vegetable waste exhibited reduced myristic acid content, with the lowest level (3.2 g/100 g larvae) observed in larvae that received conventional cucumber and ecological tomato wastes. These dietary adjustments led to an overall increase in the polyunsaturated/saturated fatty acid ratio in all larvae that received vegetable supplementation (0.5–0.64) compared to those on the control diet (0.81) (Table 5), which was also reported in studies using orange albedo as a supplementary source in the diet [26]. It is worth noting that the total fat content of the larvae is also impacted by the provided diet, resulting in a significant 13% reduction when cucumber from either conventional or ecological farming is used as a dietary supplement.

The lipid composition in the larvae is influenced by the dietary intake, with a stronger connection to the content of non-fibrous carbohydrates, starch, and protein in the feeding substrates than their fatty acid content [12,37]. The biosynthesis pathway that converts myristic acid into linoleic acid involves a series of enzymatic reactions, including chain elongation and desaturation, with several additional enzymes being involved. It is likely that the larvae adapt their metabolism to the available diet to optimize energy preservation or acquisition. This suggests that Acetyl-CoA carboxylase and fatty acid synthase may play key roles in the de novo synthesis of fatty acids from carbohydrates. Zhou et al. [38] reported the presence of Δ^9−^desaturase^18^ and Δ^12−^desaturase producing linoleic acid from oleic acid in *Acheta domesticus*, enzymes that could be overexpressed under this situation. It should also be noted that elaidic acid (C18:1n9t) instead of oleic acid (C18:1n9c) was identified in larvae, which requires further research to determine whether the presence of the trans isoform of the C18:1 fatty acid (generally associated with health effects in its cis isoform) could have any negative health consequences if consumed in large amounts. Therefore, some metabolic changes could be triggered by supplementation, although further enzyme expression and activity studies would be necessary to better understand this modulation.

To the best of our knowledge, no studies have compared the effects of using conventional or ecological vegetable waste as dietary supplements for insects on the nutritional composition of larvae, including their fatty acid contents and proportions. However, despite the fact that it could be hypothesized that the differences in micronutrients and bioactive compounds between these waste sources might influence the larval composition, our results have not shown big differences, showing that both conventional and ecological cucumber and tomato waste can be utilized as supplements to enhance the proportion of linoleic acid and the polyunsaturated/saturated fatty acid ratio in larvae. These changes in the fatty acid profile have been associated with a reduced risk of certain cardiovascular diseases [34].

High values of the PUFA/SFA ratio (>1) are correlated with a significant positive effect on cardiovascular health [20,39]. According to the obtained results, the PUFA/SFA ratio values were higher in larvae fed with supplemented diets than those of larvae fed with just wheat bran (Table 6). The supplementation of cucumber and tomato wastes (regardless of the type of harvesting) in the larvae diet improves the PUFA/SFA ratio, which increases the nutritional value of larvae. However, no significant differences were detected among feeding larvae with tomato or cucumber wastes. Besides, food characterized by a low value of IA can reduce the levels of total cholesterol and LDL-C in human blood plasma [20]. IA was reduced in larvae when they were fed with vegetable wastes as diet supplements. The obtained values of IA were higher than those obtained in *T. molitor* larvae fed with *M. oleifera* leaves, a great source of antioxidants [16]. However, similar values have been reported in green and red seaweed, milk, and other dairy products [20]. The HPI is the inverse of IA, and it has been reported that the higher the value, the better for human health [20]. The HPI value was low, which is highly influenced by a high concentration of myristic acid (C14:0) determined in the studied larvae. The consumption of food products with a low index of thrombogenicity (IT) is also beneficial to human health [40]. The IT values of larvae subjected to diets containing cucumber or tomato wastes were lower than those of larvae fed just with wheat bran. The obtained IT values are higher than those reported by Kotsou et al. [16] for *T. molitor* larvae fed with leaves of *M. oleifera* or values reported by Pérez-Santaescolástica et al. [39]. Nevertheless, these values are lower than those obtained for traditional and novel food, such as meat, dairy products, and green algae [20]. The HH index is the ratio of hypocholesterolemic/hypercholesterolemic and it is described as a health promotion index to assess the nutritional value of dietary fat [41]. The values of the HH index obtained for *T. molitor* larvae were slightly higher than those of *T. molitor* fed with antioxidant substrates (*M. oleifera* leaves) and similar to those reported for meat and fish [20]. High COX value means greater oxidative stability and a longer shelf life. In this study, the COX values evaluated for the *T. molitor* larvae fed with diets supplemented with cucumber and tomato wastes were close to 4 (Table 6), similar to those obtained by Kotsou et al. [16]. These COX values were higher than those obtained for food oils, such as olive oil, palm oil, and fish oil [42]. However, the values are lower than those obtained for sunflower oil (around 6) and canola oil (around 4.6) [43,44]. Considering all of the above, supplementing the *T. molitor* larvae with cucumber and tomato wastes (both from conventional and ecological farming) improves most of the nutritional indices assessed (Table 6), leading to a higher nutritional value of the larvae.

### 3.3. Impact of Diet on Feed Conversion Efficiency and Environmental Resource Utilization

#### 3.3.1. Feed Conversion Efficiency

**Figure 3:**
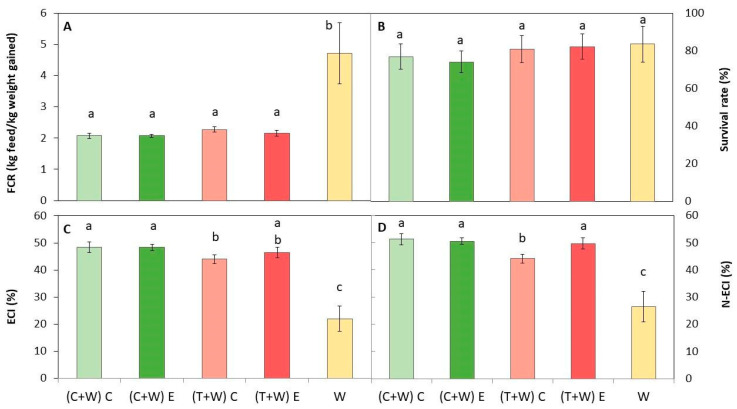
Growth performance of *T. molitor* after rearing with different diets [(C + W) C: cucumber waste + wheat bran, conventional crop; (C + W) E: cucumber waste + wheat bran, ecological crop; (T + W) C: tomato waste + wheat bran, conventional crop; (T + W) E: tomato waste + wheat bran, ecological crop; W: wheat bran]. (**A**) Feed conversion rate (FCR); (**B**) survival rate (%); (**C**) efficiency of conversion of ingested feed (ECI); and (**D**) nitrogen conversion efficiency (N-ECI). Different letters indicate statistical difference (*p* < 0.05) among the larvae fed with different diets. Values are expressed as mean ± standard deviation (*n* = 5).

Diets were composed of the macronutrient ratio recommended for optimal growth of *T. molitor*: 61.3–79.2% for carbohydrates, 14.4–25.4% for protein, and 4.3–13.4% for fat [13,28]. All diets were accepted by mealworms since the survival rate was over 74%, regardless of the provided diet (Figure 3), with values similar to those reported by other authors [11,45]. Therefore, all diets were adequate for efficiently rearing larvae.

On the other hand, feed conversion ratios of larvae, FCR, ECI, and N-ECI, significantly differed depending on the provided diet (Figure 3). The lowest FCR was obtained for those insects fed with supplemented diets, which was similar regardless of the type of waste (cucumber or tomato) and the kind of harvesting (conventional or ecological). The lower the FCR, the lower the quantity of feed necessary to obtain one kg of larvae. Conversely, the higher the ECI, the better the conversion of ingested feed by insects, which is inversely proportional to FCR. Thus, the highest ECI and N-ECI were obtained in supplemented larvae, being slightly superior in larvae fed with (C + W) C, (C + W) E, and (T + W) E (Figure 3). In addition, the N-ECI was higher than the ECI values, which suggests that nitrogen was more efficiently assimilated than other macronutrients.

Therefore, supplementing the conventional diet of larvae with vegetable wastes was more efficient than rearing *T. molitor* with just wheat bran. Generally, insects showed a better feed conversion ratio compared with other livestock since they do not need to maintain their body temperature. Specifically, the FCR has been reported to be similar to that of poultry meat (FCR = 2.3) [11], and similar values have been reported by other authors when using wheat bran as feed [12,45].

A relationship between diet composition and feed conversion ratios has been described in previous studies [11,12]. The protein content of substrates has been suggested to strongly influence FCR and ECI values, whereas in other animals, the provided energy (kcal) is what affects growing the most as it is used to maintain their body temperature [46]. Thus, higher protein availability in the diet reduces the FCR and increases the ECI values. Some researchers have pointed out that the nitrogen content and the ratio of carbohydrates to protein in the diet also play a crucial role in the development time and growth of *T. molitor* and other insects. Nitrogen is essential for protein synthesis and tissue growth, so a lower nitrogen content is associated with reduced growth [47]. Rho and Lee [48] reported that the protein/carbohydrate is 1:1 for optimal growth. However, while protein content may be relevant, our results show that it is not the only factor affecting *T. molitor* growth. In the present study, the protein content was similar among diets, although micronutrients or bioactive compounds may promote growth or decrease mortality, as proposed by Zhang et al. [31]. By supplementing the traditional wheat bran diet with cucumber or tomato waste (both from conventional and ecological cultivation), the available nitrogen was slightly increased, along with additional minerals (such as potassium, copper, sulfur, and chlorine), vitamins (particularly vitamin B5 and B6 from cucumber waste, and vitamins C and E from tomato waste) (Table 2), and other bioactive compounds like carotenoids and phenolic compounds (Table 2), which may accelerate larval growth [22].

Furthermore, our results suggest that the presence of moisture in the diet is of great importance for the performance of *T. molitor*, as previously observed [24]. Therefore, other factors can affect the growing efficiency of larvae, such as the method of providing water. Oonincx et al. [11] reported that the use of carrot as a water supply in low-protein (about 13.6% dry matter) diets increased the FCR, whereas in high-protein diets (about 22.4% dry matter), the FCR was similar to that obtained in control diets (about 17.3% dry matter). It is possible that the water contained in the agricultural waste was better assimilated by the larvae compared to the water added to the wheat bran control diet, which may have evaporated before being retained. This hypothesis is supported by the moisture of larvae at the end of the study, since those fed with just wheat bran plus water had a moisture content of 56.85%, whereas those supplemented presented a higher percentage (65.34–67.08%) (Table 4). No discernible differences were found in the studied growth parameters between cucumber and tomato waste, nor between conventional and ecological crops. Therefore, all of these types of vegetable waste can be effectively utilized for insect bioconversion as promoters of larval growth.

#### 3.3.2. Reduction in Agricultural Wastes and Water Use by Supplementing Insect Diets

The amount of commodities and water necessary to generate 1 kg of *T. molitor* larvae depended on the provided diet (Table 7). In wheat bran diets (control), it was necessary to provide 3.82 L of water, while supplementation with vegetable wastes did not require adding water to the breeding trays since they provided their own moisture to the larvae. Regarding the amount of feed necessary to produce 1 kg of larvae, supplementation of the control diet at a 1:1 (*w*/*w*) ratio with vegetable wastes allows for the reduction in the quantity of wheat bran between 43 and 47% (without observing significant differences between the vegetable supplements used). Therefore, it is noteworthy that the total water and dry matter provided in all supplemented diets were almost half of those provided in the control diet (Table 7). As a result, the total quantity of larvae generated during the 6 weeks of study was doubled when they were supplemented with cucumber and tomato wastes, regardless of the type of harvesting (conventional or ecological), as has been previously explained in Section 3.1.

Contrary to what was observed for the production of larvae, the quantity of commodities and water necessary to generate 1 kg of frass was slightly influenced by the type of the provided diet, requiring around 1.26 kg of wheat bran and a similar quantity of vegetable waste or water (Table 8). Supplementation with vegetable waste did not require the provision of additional water, unlike diets just based on wheat bran (control). The quantity of wheat bran and vegetable wastes to obtain 1 kg of frass was similar regardless of the type of diet. Thus, the total frass quantity generated by larvae over the six weeks of study was similar (around 5.4–6.0 kg) in all of the studied groups.

Therefore, depending on the target bioproduct, insect flour or biofertilizer, the selection of the optimal diet will differ. For instance, to produce insect flour, for which larvae are required, a diet supplemented with vegetable waste will be more profitable, while a similar amount of frass, with the potential to be used as biofertilizer, will be obtained with all of the studied diets.

All of the above calculations support that supplementing *T. molitor’s* diet with vegetable wastes is not only effective for the growing performance, but also allows us to reduce the amount of wheat bran and added water required, which leads to great economic savings in raw materials and water consumption in insect farms. In parallel, this strategy is also successful in reducing the greenhouse gases generated by the accumulation of agri-food wastes and the consumption of energy resources by reducing the rearing time.

## 4. Conclusions

The utilization of cucumber or tomato wastes from both conventional and ecological cultivation practices has demonstrated the potential to significantly reduce the rearing time of *T. molitor* larvae by 3–4 weeks. This fact entails notable energy, water, and cost savings in insect farms while reducing negative environmental impacts. The supplemented larvae attain a total protein content of around 50% (*w*/*w*) FW and exhibit an improved fatty acid profile characterized by higher proportions of polyunsaturated fatty acids (linoleic acid). Therefore, feeding *T. molitor* larvae with such vegetable wastes enhances their nutritional and health profile, making them a promising source of high-quality protein and healthy fatty acids for human or animal consumption.

Despite the differences in the nutritional composition and content of bioactive compounds of the supplements due to the type of vegetable used (cucumber or tomato) or cultivation style (conventional or organic), the results of all supplemented groups were very similar, which suggests that the provision of water as part of these supplements instead of being sprayed on the base substrate (wheat bran in this study) may be the main cause of the improvements observed with supplementation.

## Figures and Tables

**Table 1:** Nutritional composition of commodities (vegetable wastes and wheat bran) used for feeding *T. molitor* larvae.

Commodities	Moisture % (*w*/*w* FW)	Total Protein Content % (*w*/*w* FW)	Total Carbohydrates Content % (*w*/*w* FW)	Total Fat Content % (*w*/*w* FW)	Ash % (*w*/*w* FW)
Conventional cucumber	94.76 ± 0.19 a	0.71 ± 0.05 a	4.07 ± 0.23 a	0.18 ± 0.03 a	0.28 ± 0.02 a
Ecological cucumber	95.56 ± 0.11 b	0.72 ± 0.03 a	3.22 ± 0.12 b	0.18 ± 0.02 a	0.32 ± 0.02 ab
Conventional tomato	92.02 ± 0.25 c	1.05 ± 0.03 b	6.26 ± 0.29 c	0.23 ± 0.02 a	0.45 ± 0.04 b
Ecological tomato	90.54 ± 0.60 d	0.95 ± 0.06 ab	7.81 ± 0.64 d	0.24 ± 0.02 a	0.46 ± 0.02 b
Wheat bran	8.41 ± 0.20 e	15.07 ± 0.29 c	66.90 ± 0.37 e	5.20 ± 0.18 b	4.42 ± 0.20 c

Different letters indicate significant differences (*p* < 0.05) among moisture and macronutrients of commodities. FW: fresh weight.

**Table 2:** Vitamins and bioactive compounds content of commodities used for feeding *T. molitor* larvae.

Commodities	Water-Soluble Vitamins (mg/100 g FW)						Fat-Soluble Vitamins (mg/100 g FW)		Bioactive Compounds	
	C	B1	B2	B3	B5	B6	E	K	Total Phenolic Compounds (mg Eq GA/100 g FW)	Total Carotenoids (mg/100 g FW)
Conventional cucumber	<0.1 a	0.056 ± 0.047 a	0.066 ± 0.007 a	0.116 ± 0.006 a	0.253 ± 0.127 a	0.214 ± 0.056 a	0.070 ± 0.043 a	0.0040 ± 0.0026 a	20.67 ± 0.34 a	3.456 ± 0.267 a
Ecological cucumber	<0.1 a	0.027 ± 0.017 a	0.038 ± 0.007 a	0.119 ± 0.022 a	0.238 ± 0.197 a	0.227 ± 0.035 a	0.060 ± 0.035 a	0.0052 ± 0.0037 a	16.03 ± 3.59 b	1.907 ± 0.181 a
Conventional tomato	16.33 ± 7.64 b	0.053 ± 0.012 a	0.052 ± 0.032 a	0.119 ± 0.057 a	0.097 ± 0.064 a	0.073 ± 0.033 ab	0.236 ± 0.024 b	0.0037 ± 0.0011 a	79.38 ± 0.81 c	289.07 ± 49.90 b
Ecological tomato	21.00 ± 8.00 b	0.050 ± 0.035 a	0.142 ± 0.044 b	0.114 ± 0.061 a	0.074 ± 0.055 a	0.049 ± 0.039 b	0.311 ± 0.010 b	0.0056 ± 0.0020 a	83.87 ± 0.21 d	256.75 ± 21.53 b
Wheat bran	<0.1 a	0.533 ± 0.051 b	0.252 ± 0.026 c	0.587 ± 0.042 b	2.523 ± 0.402 b	0.217 ± 0.173 a	1.005 ± 0.098 c	0.0049 ± 0.0038 a	128.01 ± 1.95 e	0.081 ± 0.009 a

Different letters indicate significant differences (*p* < 0.05) among vitamins and bioactive compounds content of commodities. FW: fresh weight; Eq GA: gallic acid equivalent.

**Table 3:** Quantity of water and macronutrients provided to *T. molitor* larvae for each diet condition.

Diet	Total Water (kg)	Total Protein Content (kg)	Total Carbohydrate Content (kg)	Total Fat Content (kg)	Ash (kg)
(C + W) C	1.444 ± 0.003 a	0.220 ± 0.004 a	0.993 ± 0.006 a	0.075 ± 0.002 a	0.066 ± 0.003 ab
(C + W) E	1.455 ± 0.008 b	0.221 ± 0.004 a	0.981 ± 0.011 a	0.075 ± 0.002 a	0.066 ± 0.003 ab
(T + W) C	1.405 ± 0.008 c	0.225 ± 0.004 a	1.024 ± 0.010 b	0.076 ± 0.002 a	0.068 ± 0.003 b
(T + W) E	1.385 ± 0.001 d	0.224 ± 0.004 a	1.045 ± 0.004 c	0.076 ± 0.002 a	0.068 ± 0.003 b
W	1.517 ± 0.024 e	0.210 ± 0.005 b	0.936 ± 0.017 d	0.073 ± 0.002 a	0.062 ± 0.003 c

Different letters indicate significant differences (*p* < 0.05) among diets. [(C + W) C: cucumber waste + wheat bran, conventional crop; (C + W) E: cucumber waste + wheat bran, ecological crop; (T + W) C: tomato waste + wheat bran, conventional crop; (T + W) E: tomato waste + wheat bran, ecological crop; W: wheat bran].

**Table 4:** Macronutrient composition of *T. molitor* larvae fed with different diets.

Diet	Moisture % (*w*/*w* FW)	Total Protein Content % (*w*/*w* FW)	Total Carbohydrate Content % (*w*/*w* FW)	Total Fat Content % (*w*/*w* FW)	Ash % (*w*/*w* FW)
(C + W) C	65.34 ± 0.81 a	49.73 ± 0.19 a	17.98 ± 0.31 a	24.44 ± 0.37 a	4.15 ± 0.06 a
(C + W) E	65.63 ± 0.40 a	49.25 ± 0.31 b	17.33 ± 0.06 ab	24.76 ± 0.07 a	3.96 ± 0.04 b
(T + W) C	67.08 ± 0.29 b	49.1 ± 0.18 b	15.93 ± 1.36 b	27.17 ± 1.82 ab	3.94 ± 0.07 b
(T + W) E	66.16 ± 0.52 a	50.19 ± 0.33 a	14.31 ± 0.16 c	27.07 ± 0.47 ab	3.97 ± 0.03 b
W	56.85 ± 0.05 c	45.58 ± 0.22 c	18.17 ± 1.19 a	28.20 ± 2.06 b	4.25 ± 0.04 c

Different letters indicate significant differences (*p* < 0.05) among larvae fed with different diets. [(C + W) C: cucumber waste + wheat bran, conventional crop; (C + W) E: cucumber waste + wheat bran, ecological crop; (T + W) C: tomato waste + wheat bran, conventional crop; (T + W) E: tomato waste + wheat bran, ecological crop; W: wheat bran].

**Table 5:** Fatty acid composition of *T. molitor* larvae fed with different diets.

Fatty Acids (g/100 g FW)		Diet				
		(C + W) C	(C + W) E	(T + W) C	(T + W) E	W
Lauric acid	C12:0	0.414 ± 0.059 a	0.706 ± 0.17 ab	0.694 ± 0.196 ab	0.442 ± 0.06 a	0.968 ± 0.247 b
Tridecanoic acid	C13:0	0.010 ± 0.001 a	0.015 ± 0.002 a	0.015 ± 0.003 ab	0.009 ± 0.001 a	0.021 ± 0.007 b
Myristic acid	C14:0	3.20 ± 0.24 a	3.62 ± 0.04 ab	4.02 ± 0.38 b	3.24 ± 0.24 a	5.98 ± 0.59 c
Myristoleic acid	C14:1	0.479 ± 0.03 a	0.534 ± 0.01 a	0.623 ± 0.108 a	0.450 ± 0.024 a	0.934 ± 0.183 b
Pentadecylic acid	C15:0	0.042 ± 0.002 a	0.031 ± 0.009 b	0.047 ± 0.005 a	0.037 ± 0.005 ab	0.033 ± 0.002 b
Palmitic acid	C16:0	3.24 ± 0.03 a	3.64 ± 0.07 b	3.72 ± 0.11 b	3.34 ± 0.02 a	3.56 ± 0.22 b
Palmitoleic acid	C16:1	0.204 ± 0.01 a	0.218 ± 0.004 ab	0.234 ± 0.016 b	0.214 ± 0.005 ab	0.313 ± 0.017 c
Stearic acid	C18:0	1.27 ± 0.14 a	1.16 ± 0.07 a	1.22 ± 0.11 a	1.51 ± 0.15 b	1.19 ± 0.12 a
Elaidic acid	C18:1n9t	5.43 ± 0.15 a	5.47 ± 0.01 a	5.82 ± 0.43 ab	6.27 ± 0.16 bc	6.58 ± 0.58 c
Linoleic acid	C18:2n6c	9.71 ± 0.04 a	8.95 ± 0.19 b	10.35 ± 0.08 c	11.10 ± 0.06 d	8.41 ± 0.11 e
Linoleic acid	C18:3n3	0.138 ± 0.001 a	0.127 ± 0.001 b	0.135 ± 0.003 ab	0.137 ± 0.012 a	0.094 ± 0.002 c
Araquidonic acid	C20:4n6	0.345 ± 0.03 a	0.336 ± 0.023 a	0.340 ± 0.029 a	0.337 ± 0.071 a	0.280 ± 0.01 a
Saturated fatty acids		8.50 ± 0.46 a	9.71 ± 0.16 bc	10.34 ± 0.47 c	9.04 ± 0.15 ab	12.68 ± 0.69 d
Monounsaturated fatty acids		5.78 ± 0.39 a	5.69 ± 0.01 a	6.05 ± 0.41 ab	6.49 ± 0.16 bc	6.89 ± 0.57 c
Polyunsaturated fatty acids		10.19 ± 0.07 a	9.41 ± 0.17 b	10.82 ± 0.05 c	11.57 ± 0.01 d	8.78 ± 0.1 e
Total fatty acids (% *w*/*w* FW)		24.44 ± 0.37 a	24.76 ± 0.07 a	27.17 ± 1.82 ab	27.07 ± 0.47 ab	28.20 ± 2.06 b

Different letters indicate significant differences (*p* < 0.05) among larvae fed with different diets. [(C + W) C: cucumber waste + wheat bran, conventional crop; (C + W) E: cucumber waste + wheat bran, ecological crop; (T + W) C: tomato waste + wheat bran, conventional crop; (T + W) E: tomato waste + wheat bran, ecological crop; W: wheat bran]. Concentration of fatty acids expressed as g/100 g FW. FW: fresh weight.

**Table 6:** Calculated nutritional indices of *T. molitor* larvae fed with different diets.

Diet	PUFA/SFA	IA	IT	HH	HPI	COX
(C + W) C	1.25 ± 0.04 a	1.01 ± 0.09 ab	0.91 ± 0.03 a	2.29 ± 0.16 a	1.00 ± 0.09 a	4.43 ± 0.03 a
(C + W) E	1.03 ± 0.04 b	1.21 ± 0.02 c	1.03 ± 0.01 b	1.87 ± 0.05 b	0.83 ± 0.01 b	4.05 ± 0.010 b
(T + W) C	1.11 ± 0.05 c	1.17 ± 0.13 bc	0.99 ± 0.04 b	1.98 ± 0.19 b	0.86 ± 0.10 b	4.24 ± 0.05 c
(T + W) E	1.35 ± 0.02 d	0.91 ± 0.07 d	0.84 ± 0.01 a	2.54 ± 0.14 c	1.11 ± 0.09 a	4.56 ± 0.04 d
W	0.75 ± 0.05 e	1.72 ± 0.022 e	1.26 ± 0.08 c	1.47 ± 0.18 d	0.59 ± 0.08 c	3.38 ± 0.07 e

Different letters indicate significant differences (*p* < 0.05) among larvae fed with different diets. [(C + W) C: cucumber waste + wheat bran, conventional crop; (C + W) E: cucumber waste + wheat bran, ecological crop; (T + W) C: tomato waste + wheat bran, conventional crop; (T + W) E: tomato waste + wheat bran, ecological crop; W: wheat bran]. Nutritional indices: IA: index of atherogenicity; IT: index of thrombogenicity; HH: hypocholesterolemic/hypercholesterolemic ratio; HPI: health-promoting index; COX: calculated oxidizability value.

**Table 7:** Consumption of water and commodities necessary for an optimal growing performance of *T. molitor* to obtain 1 kg of larvae and total quantity of larvae generated over the 6 weeks.

Diet	Added Water (L/kg Larvae)	Wheat Bran (kg/kg Larvae)	Vegetable Waste (kg/kg Larvae)	Total Water Provided in Diet (L/kg Larvae)	Total Dry Matter Provided in Diet (kg/kg Larvae)	Total Larvae Generated (kg)
(C + W) C	-	1.91 ± 0.07 a	1.91 ± 0.07 a	2.04 ± 0.07 a	1.79 ± 0.06 a	3.66 ± 0.02 a
(C + W) E	-	1.92 ± 0.04 a	1.92 ± 0.04 a	2.06 ± 0.04 a	1.79 ± 0.04 a	3.64 ± 0.02 a
(T + W) C	-	2.03 ± 0.06 a	2.03 ± 0.06 b	2.12 ± 0.06 a	1.95 ± 0.06 a	3.46 ± 0.02 a
(T + W) E	-	1.93 ± 0.07 a	1.93 ± 0.07 a	2.00 ± 0.07 a	1.86 ± 0.07 a	3.63 ± 0.02 a
W	3.82 ± 0.58	3.82 ± 0.58 b	0.00 ± 0.00 c	4.24 ± 0.64 b	3.40 ± 0.52 b	1.87 ± 0.06 b

Different letters indicate statistical difference (*p* < 0.05) between the consumed resources in each diet. Values are expressed as mean ± standard deviation (*n* = 5).

**Table 8:** Consumption of water and commodities necessary for an optimal growing performance of *T. molitor* to obtain 1 kg of frass and quantity of frass generated over the 6 weeks.

Diet	Added Water (L/kg Frass)	Wheat Bran (kg/kg Frass)	Vegetable Waste (kg/kg Frass)	Total Water Provided in Diet (L/kg Frass)	Total Dry Matter Provided in Diet (kg/kg Frass)	Total Frass Generated (kg)
(C + W) C	-	1.28 ± 0.06 a	1.28 ± 0.06 a	1.36 ± 0.06 a	1.20 ± 0.05 a	5.48 ± 0.25 a
(C + W) E	-	1.23 ± 0.02 abc	1.23 ± 0.02 ab	1.32 ± 0.02 a	1.14 ± 0.02 a	5.70 ± 0.09 a
(T + W) C	-	1.20 ± 0.05 bc	1.20 ± 0.05 b	1.25 ± 0.05 a	1.15 ± 0.05 a	5.84 ± 0.24 a
(T + W) E	-	1.18 ± 0.06 c	1.18 ± 0.06 b	1.22 ± 0.06 a	1.14 ± 0.05 a	5.94 ± 0.28 a
W	1.26 ± 0.04	1.26 ± 0.04 ab	0.00 ± 0.00 c	1.4 ± 0.05 a	1.12 ± 0.04 a	5.56 ± 0.18 a

Different letters indicate statistical difference (*p* < 0.05) between the consumed resources in each diet. Values are expressed as mean ± standard deviation (*n* = 5).

## Data Availability

Data are contained within the article.
